# Cancer symptom awareness and barriers to medical help seeking in Scottish adolescents: a cross-sectional study

**DOI:** 10.1186/1471-2458-14-1117

**Published:** 2014-10-29

**Authors:** Gill Hubbard, Iona Macmillan, Anne Canny, Liz Forbat, Richard D Neal, Ronan E O’Carroll, Sally Haw, Richard G Kyle

**Affiliations:** Cancer Care Research Centre, School of Health Sciences, University of Stirling, FK9 4LA Stirling, UK; Teenage Cancer Trust, 93 Newman Street, W1T 3EZ London, UK; North Wales Centre for Primary Care Research, Bangor University, LL13 7YP Bangor, UK; Division of Psychology, School of Natural Sciences, University of Stirling, FK9 4LA Stirling, UK; School of Health Sciences, University of Stirling, FK9 4LA Stirling, UK

**Keywords:** Public cancer awareness, Early diagnosis, Help seeking behaviour, Adolescents

## Abstract

**Background:**

Initiatives to promote early diagnosis include raising public awareness of signs and symptoms of cancer and addressing barriers to seeking medical help about cancer. Awareness of signs and symptoms of cancer and emotional barriers, such as, fear, worry, and embarrassment strongly influence help seeking behaviour. Whether anxiety influences seeking medical help about cancer is not known. The purpose of this study about adolescents was to examine: 1) the relationship between contextual factors and awareness of signs and symptoms of cancer and barriers (including emotional barriers) to seeking medical help, and 2) associations between anxiety and endorsed barriers to seeking medical help. Interpretation of data is informed by the common sense model of the self-regulation of health and illness.

**Methods:**

A cross-sectional study of 2,173 Scottish adolescents (age 12/13 years) using the Cancer Awareness Measure. Socio-demographic questions were also included. Descriptive statistics were calculated and two Poisson regression models were built to determine independent predictors of: 1) the number of cancer warning signs recognized, and; 2) number of barriers to help seeking endorsed.

**Results:**

Analysis identified that knowing someone with cancer was a significant independent predictor of recognising more cancer warning signs whereas Black and Minority Ethnic status was a significant independent predictor of recognising fewer cancer warning signs. Emotional barriers were the most commonly endorsed, followed by family, service and practical barriers. Over two thirds of adolescents were ‘worried about what the doctor would find’ and over half were ‘scared’. Higher anxiety scores, knowing more cancer warning signs and female gender were significant independent predictors of barriers to help seeking.

**Conclusion:**

Improving cancer awareness and help seeking behaviour during adolescence may contribute to early presentation. Contextual factors (for example, ethnicity, gender, knowing someone with cancer), and emotional dimensions (for example, anxiety, fear, worry) are critical components in help seeking behaviours. The role of emotional factors indicates that public health campaigns focused on awareness and help seeking may benefit from having a more emotional focus, for example, including references to feelings, such as, fears and worries.

## Background

### Promoting early presentation

Cancer is the leading cause of non-accidental death in teenagers and young people (10–19 years) [1]. In the United Kingdom (UK) there are around 2,200 teenagers and young people (15–24 year olds) diagnosed each year and more than 80% survive the disease for at least 5 years, although there is considerable variation in survival between diagnostic groups [2]. Although the importance of early diagnosis in relation to survival is uncertain [3, 4], there is sufficient evidence for UK governments to commit to improving survival by increasing the proportion of people with early diagnosis [5–8]. Initiatives to promote early diagnosis include addressing symptom appraisal and help seeking intervals by raising public awareness of signs and symptoms of cancer and addressing barriers to seeking medical help about cancer [5, 8, 9].

Lower recognition of cancer warning signs is linked to delays in seeking medical help [10, 11]. Not recognising a symptom as suspicious is one of the most common reasons given by patients with cancer for delays in seeking medical help [12, 13]. Population-based studies show that adult and adolescent awareness of signs and symptoms of cancer is low [14–19]. Evidence suggests that awareness is lower among males and adults in ‘lower’ occupational groups and ethnic minority groups [14]. These groups in particular, therefore, may be at risk of presenting later with symptoms. There is, however, only limited evidence about demographic variations in cancer awareness among adolescents [15, 16] and consequently limited evidence about who in this age group is at risk of not presenting early. Kyle and colleagues found that girls compared to boys and ethnic minority compared to White adolescents recognised fewer warning signs for cancer but these differences were not statistically significant [15]. They also found that ‘knowing someone with cancer’ was associated with recognition of more warning signs for cancer and endorsement of more barriers to seeking medical help [15]. There remains a level of uncertainty therefore about the relationship between contextual factors (for example, ethnicity, gender, knowing someone with cancer) and awareness of signs and symptoms of cancer during adolescence. Studies involving larger sample sizes may contribute towards addressing this uncertainty.

It is not simply lack of awareness of signs and symptoms of cancer that will influence help seeking behaviour [17]. Empirical evidence of barriers to seeking medical help about cancer suggests that emotional barriers, such as, fear, worry, and embarrassment strongly influence help seeking behaviour [10, 13–15, 18–21]. A qualitative synthesis of 32 international papers for instance, found strong similarities in patients with different cancer diagnoses regarding help seeking experiences and delay in cancer presentation [13]. Key themes were recognition and interpretation of symptoms and fear of consultation, with fear manifesting as a fear of embarrassment (the feeling that symptoms were trivial or that symptoms affected a sensitive body area), or a fear of cancer (pain, suffering, and death), or both [13]. de Nooijer and colleagues found that fear leads some people to promptly seek medical help about cancer and others to avoid seeking help [10]. Drawing on Levanthal’s concepts of danger and fear control, they suggest that avoidance is a coping strategy used by people to manage illness anxieties [10, 22].

There is some empirical evidence suggesting that behavioural response to managing illness anxieties is influenced by a fundamental dispositional characteristic or trait that is manifest in the degree of tendency toward anxiety, worry and negative emotions in general [23, 24]. Ristvedt and Trinkaus for instance, found that a decreased tendency toward worry was associated with delays in seeking medical help for symptoms of rectal cancer [24]. Whether anxiety is related to seeking medical help for different types of cancer or during adolescence, however, is not known.

Taken together, the empirical evidence suggests that anxieties, worries and fears will influence seeking medical help about cancer and studies of emotional dimensions of help seeking have been recommended [25]. Theories that focus on emotional dimensions of symptom appraisal and help seeking may therefore be particularly helpful in interpreting empirical evidence. No single psychological theory or model is likely to explain behaviour in response to symptoms [17, 25–28]. Given the strength of empirical evidence reporting the influence of anxieties, worries and fears on seeking medical help about cancer, it seems reasonable to focus on models that include these emotional processes. A recent review of three models of illness behaviour in response to symptoms found that only the Common Sense Model of the Self-Regulation of Health and Illness considers the role of emotions in response to illness [28].

According to the common sense model, individuals create mental representations of symptoms [29–31]. When an individual experiences a threat to health (for example, signs and symptoms of cancer) he or she will actively process the meaning of somatic stimuli and generate two sets of representations – cognitive representations or interpretations of the nature of the threat, and emotional representations, such as, fear [32]. These representations generate parallel but reciprocal behavioural attempts at regulation of the threat itself and regulation of the emotions engendered by it [32].

The past decade has witnessed a growth in emotion regulation research [33, 34]. Cameron and Jago for instance, have expanded the common sense model by delineating four coping strategies to regulate emotions including, avoiding or focusing on the threat, and proactive behaviours to reduce the threat [35]. Hence, according to the common sense model, emotional representations of signs and symptoms of cancer (for example, fear and worry) will influence the behavioural response (for example, seeking or avoiding seeking medical help) to regulate the health threat (cancer) *and* to regulate illness anxiety [35]. Thus, barriers to seeking medical help about cancer, such as ‘I would be worried about what the doctor might find’ may symbolise an intentional behavioural response (avoidance) to managing illness anxieties; i.e., anxieties evoked by the threat of cancer [14, 15, 20].

The common sense model is also a useful conceptual framework because it recognises the role of significant others. As described above, ‘knowing someone with cancer’ was associated with higher recognition of signs and symptoms of cancer [15]. According to the common sense model, illness representations are guided by three basic sources of information, including information from the external social environment, such as information from perceived significant others [36, 37]. Illness representations are influenced by a range of factors including knowing someone else with experience of the illness, information from friends and relatives, and the media [36]. The model proposes that significant others will influence for instance, an individual’s beliefs about the extent to which a disease can be *cured or controlled*, the *cause* of a disease and the *consequences* of the disease to a person’s life [29]. It is these beliefs that may influence an individual’s emotional representation of cancer (for example, fear and worry) [35].

To address gaps in evidence the aim of this study about adolescents was therefore to examine: 1) the relationship between contextual factors (gender, ethnicity, deprivation, knowing someone with cancer) and awareness of signs and symptoms of cancer and barriers (including emotional barriers) to seeking medical help during adolescence, and 2) associations between anxiety and endorsed barriers to seeking medical help during adolescence. In doing so, we aim to provide insight into factors likely to influence symptom appraisal and help seeking intervals during adolescence and inform further research about early presentation [9]. The study focused on early adolescence (12/13 years) because it is the start of adolescence, which is a key life stage transition.

## Methods

### Study design

Data were drawn from the Adolescent Cancer Education (ACE) study, the design of which is described in the published protocol [38]. Briefly, ACE is a cluster randomised controlled trial (RCT) to assess the effectiveness of a school-based educational intervention on adolescents’ and parents’ cancer awareness. This paper reports cross-sectional analysis of adolescents’ baseline data.

### Setting and Sample

All 29 state High Schools (excluding 44 Additional Support for Learning schools) in the Glasgow City Council area were invited to participate by a letter of invitation (see section below); 20 schools (69.0%) were recruited. Nine schools either informed a researcher that they did not wish to participate (n = 3) or after three attempts to speak with the head-teacher by telephone were unable to be reached (n = 6). To the best of our knowledge the composition of non-participating schools was not systematically different from participating schools since non-participating schools exhibited a similar geographical spread and deprivation profile to participating schools.

**Figure 1:**
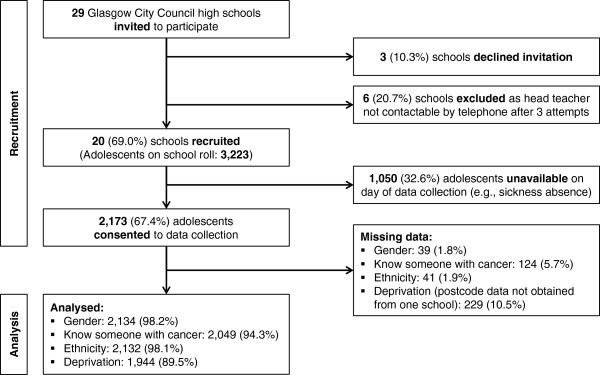
**Participant Flowchart.**

There were 3,223 adolescents on the school register at the end of their first year (S1) of education (age 12/13 years) in study schools; 2,173 (67.4%) consented to data collection. We do not know how many students were in attendance on the days consent was given. Thus, we are uncertain if the sample reflects school absences on the day students were consenting, or whether some classes in S1 were not given the opportunity to complete the survey, or whether some students did not consent. A study flowchart is shown in Figure 1.

### Recruitment and consent

Schools were recruited in May 2013 and adolescents in June 2013. School head-teachers were contacted by letter, which was followed up with a telephone call and face-to-face meeting to invite participation in the study. Parents/carers were sent a letter and information sheet about the study, which included a form to be returned to school if they wished to opt their child out of the study. The opt-out method of parental consent has been found to be ethically acceptable [39]. No parent/carer refused to allow their child to participate in the study. Adolescents were provided with an information sheet about the study at the time measurements were undertaken and also asked to give written consent to their participation in the study.

### Survey instrument

Data were collected using a self-complete paper questionnaire administered by teachers to a whole class under exam conditions but students were informed that it was not a test. Teachers encouraged students to complete as much of the questionnaire as they could within the 50 or 55 minute lesson period. The instrument incorporated the Cancer Awareness Measure (CAM) [40], and socio-demographic questions.

#### Cancer awareness

Adolescent cancer awareness was measured using CAM items. This instrument has been used in previous studies of adolescent cancer awareness and its content and validation for adolescents is described in detail elsewhere [15, 41]. The CAM included closed questions to measure recognition of cancer warning signs and endorsement of barriers to help seeking.

Recognition of signs and symptoms of cancer was assessed through a nine item question. The question was phrased as: ‘The following may or may not be warning signs for cancer. For example, if you think that an unexplained lump or swelling could be a sign of cancer tick the *Yes* box, if you do not think it is tick the *No* box and if you don’t know tick the *don*’*t know* box. We are interested in your opinion’. This was followed by a list of nine warning signs: lump or swelling, persistent unexplained pain, unexplained bleeding, persistent cough or hoarseness, persistent change in bowel or bladder habits, difficulty swallowing, change in the appearance of a mole, a sore that does not heal and unexplained weight loss. Responses were dichotomised for analysis (i.e., ‘Yes’ versus ‘No’/‘Don’t know’).

Barriers to help seeking were assessed with 11 items, including four emotional barriers (e.g., ‘I would be worried what the doctor might find’), three practical barriers (e.g., ‘I would too busy to make time to go to the doctor’), and three service barriers (e.g., ‘I would be worried about wasting the doctor’s time’). We also included an additional item: ‘I wouldn’t want my family to find out’ because this was included in research carried out for Scotland’s Detect Cancer Early initiative (personal communication, marketing manager at the Scottish Government). Response options were ‘Yes often’ , ‘Yes sometimes’ and ‘No’ which for analysis were re-categorised as ‘Yes’ or ‘No’. Summation of ‘Yes’ responses was used to identify a total number of barriers.

#### Socio-demographic characteristics

Socio-demographic questions were included to gather data on: age, gender and ethnicity (divided into five pre-defined categories and sub-categories): *White* (white British, white Irish, any other white background), *mixed* (white and black Caribbean, white and black African, white and Asian, any other mixed background), *Asian or Asian British* (Indian, Pakistani, Bangladeshi, any other Asian background), *black or black British* (black Caribbean, black African, any other black background), *Chinese*/*other* (Chinese, other). Students were also asked to tick ‘yes’ or ‘no’ to the following question: ‘*Have you*, *you family or close friends had cancer*?’ If they answered ‘yes’ then they were asked to indicate who had had cancer from the following list: i) you, ii) close family member (e.g. mum, dad, brother, sister, grandma, granddad), iii) other family member (e.g. aunt, uncle, cousin), iv) close friend, v) other friend.

#### Anxiety

Anxiety was assessed through items on the Hospital Anxiety and Depression Scale (HADS). HADS is a self-reported measure of anxious and depressive symptoms originally developed for use in hospital outpatient departments [42]. The instrument has since been validated for use with adolescents and is a useful diagnostic aid for identifying emotional illness in community settings [43]. HADS comprises 14 items, 7 of which relate to depression and 7 to anxiety. The anxiety scale is scored using a four-point Likert scale ranging from 0 to 3, with higher scores indicating higher incidence of emotional disorder (anxiety) The range of scores is 0 to 21. In adolescents, anxiety scores of 0 to 8 are considered normal, with a score between 9 and 11 indicating possible emotional disorder, and score above 11 indicating probable emotional disorder [43].

#### Deprivation

Adolescents’ postcodes were used to derive scores on the Scottish Index of Multiple Deprivation (SIMD) 2012 using a publically available postcode lookup tool developed by the Scottish Government [44]. SIMD is a relative measure of area-based deprivation. SIMD combines 38 indicators in 7 domains into a single index using the following weights: current income (28%), employment (28%), health (14%), education (14%), geographic access (9%), crime (5%) and housing (2%). Each of the 6,505 data zones in Scotland are ranked from 1 (most deprived) to 5 (least deprived). Quintiles are derived where 1 indicates the most deprived and 5 the least deprived areas. SIMD quintile of residence was linked to data derived from primary data collection. Due to the skewed deprivation profile of Glasgow, which includes many areas with high levels of deprivation and fewer areas with lower levels of deprivation and in accordance with the ACE study protocol [38], SIMD was dichotomised for analysis (i.e., SIMD quintile 1 [Q1 – most deprived] vs. quintiles 2 to 5 [Q2-5 – lower deprivation]).

### Data analysis

Data analysis proceeded in four stages. First, descriptive statistics were calculated for socio-demographic variables (gender, ethnicity, SIMD quintile of residence), knowing someone with cancer, HADS, and CAM items and reported as n (%) for categorical variables (e.g., gender, ethnicity) and mean (Standard Deviation [SD]) for continuous variables (e.g., number of cancer warning signs recognised, number of barriers to help seeking behaviours endorsed). Second, Pearson’s chi-square (χ^2^) tests were used to assess bivariate associations between awareness of cancer warning signs and barriers to help seeking and dichotomised socio-demographic variables: i.e., gender (Male vs. Female), knowing someone with cancer (Yes vs. No), ethnicity (White vs Black and Minority Ethnic [BME]) and deprivation (SIMD Q1 vs SIMD Q2-5). Third, independent samples t-tests were used to assess differences in the mean number of cancer warning signs recognised (out of 9) and the mean number of barriers to help seeking behaviour endorsed (out of 11) by gender, knowing someone with cancer, ethnicity and deprivation. Finally, two Poisson regression models were built to determine independent predictors of: 1) the number of cancer warning signs recognised and; 2) number of barriers to help seeking endorsed. Previous research has found an association between knowing someone with cancer and recognition of cancer warning signs [15]. Hence, in order to assess this association in a larger sample of adolescents, and for the first time adjust for deprivation, initially the following four binary variables were simultaneously entered into the model: knowing someone with cancer (Yes = 1), gender (female = 1), ethnicity (BME = 1) and deprivation (SIMD Q1 = 1). Gender was subsequently removed from the final model guided by the principle of parsimony. In order to test the hypothesis that those who experienced heightened anxiety were likely to report more barriers to help seeking a continuous variable for anxiety was included in the model initial model alongside the number of cancer warning signs recognised and the same four binary variables. Again, guided by the principle of parsimony, the binary variables knowing someone with cancer, ethnicity and deprivation were subsequently removed from the model. Data were analysed using SPSS 19.0. Significance tests were two-sided; p < 0.05 was considered statistically significant.

### Ethical considerations

Approval for the study was obtained from the Research Ethics Committee in the School of Health Sciences, University of Stirling (reference: 13/14(83)). Glasgow City Council, Planning, Performance and Research Unit approved the involvement of secondary schools. All General Practitioner practices (i.e., where primary care doctors are based) in the research site were informed about the study.

## Results

### Sample

The sample included 2,173 (female: n = 1,102, 50.7%) adolescents with a mean age of 12.4 years (SD = 0.55) at the time of the survey. SIMD data could be linked for 1,944 (89.5%) adolescents, largely due to missing postcode data from one study school. Thus, analyses including deprivation are conducted on this smaller sample of adolescents. Socio-demographic characteristics of respondents are shown in Table 1.

**Table 1:** **Sample socio**-**demographic characteristics**

	All (n = 2,173)			
	Mean	SD	%	n
**Age**	12.4	0.55		
**Gender**				
Male			47.5	1,032
Female			50.7	1,102
Missing			1.8	39
**Knew someone with cancer**				
Yes			58.3	1,266
No			36.0	783
Missing			5.7	124
**Ethnicity**				
White			84.0	1,826
BME^#^			14.1	306
Mixed			3.5	75
Asian			6.0	131
Black			2.6	57
Chinese			0.7	15
Other			1.3	28
Missing			1.9	41
**Deprivation (SIMD** ^**†**^ **)**				
Quintile 1			55.5	1,205
Quintiles 2-5			34.0	739
Missing			10.5	229
**Anxiety (HADS** ^**‡**^ **)**				
Total score (Mean (SD))			7.2	(4.19)
No emotional disorder			66.3	1,212
Possible emotional disorder			18.0	329
Probable emotional disorder			15.7	286

Notes: ^#^Black and Minority Ethnic. ^†^Scottish Index of Multiple Deprivation.

^‡^Hospital Anxiety and Depression Scale (excludes 346 cases with incomplete anxiety sub-scale data).

### Recognition of cancer warning signs

‘Unexplained lump or swelling’ was the most commonly recognised cancer warning sign (78.9%) followed by ‘change in bowel/bladder habits’ (55.2%) and ‘change in mole appearance’ (45.9%). Almost half of adolescents recognised ‘unexplained bleeding’ (44.9%), ‘persistent unexplained pain’ (44%) and ‘unexplained weight loss’ (42.4%). Just over one third recognised ‘persistent cough or hoarseness’ (34%) and ‘persistent difficulty in swallowing’ (34%). The least recognised was ‘sore that does not heal’ (26.2%). Differences by gender, knowing someone with cancer, ethnicity and deprivation for individual warning signs are shown in Table 2.

**Table 2:** **Cancer warning signs**

Cancer warning sign % Yes (n)	Gender (n = 2,134)			Knew someone with cancer (n = 2,049)			Ethnicity (n = 2,132)			Deprivation (SIMD ^†^) (n = 1,944)		
	Male (n = 1,032)	Female (n = 1,102)	Significance*	Yes (n = 1,266)	No (n = 783)	Significance*	White (n = 1,826)	BME ^#^(n = 306)	Significance*	Q1 (n = 1,205)	Q2-5 (n = 739)	Significance*
Lump or swelling	72.7 (744)	84.9 (931)	χ^2^(1, 2120) = 48.21 p < 0.001	82.5 (1040)	72.8 (567)	χ^2^(1,2040) = 27.03 p < 0.001	80.8 (1469)	68.5 (207)	χ^2^(1,2119) = 23.71 p < 0.001	79.9 (949)	77.8 (572)	χ^2^(1,1923) = 1.16 p = 0.281
Change in bowel/bladder habits	54.9 (562)	55.8 (609)	χ^2^(1,2116) = 0.17 p = 0.682	60.4 (760)	48.8 (379)	χ^2^(1,2036) = 26.18 p < 0.001	56.7 (1028)	47.5 (144)	χ^2^(1,2115) = 8.91 p = 0.003	53.3 (633)	59.2 (433)	χ^2^(1,1919) = 6.22 p = 0.013
Change in appearance of a mole	43.5 (444)	48.0 (524)	χ^2^(1,2112) = 4.38 p = 0.036	49.0 (616)	41.4 (321)	χ^2^(1,2032) = 11.10 p = 0.001	47.9 (866)	33.0 (100)	χ^2^(1,2111) = 23.20 p < 0.001	45.4 (538)	48.2 (352)	χ^2^(1,1916) = 1.38 p = 0.241
Unexplained bleeding	46.5 (476)	43.6 (475)	χ^2^(1,2113) = 1.86 p = 0.173	47.4 (596)	41.3 (320)	χ^2^(1,2033) = 7.18 p = 0.007	45.3 (821)	41.9 (126)	χ^2^(1,2112) = 1.26 p = 0.262	44.2 (523)	46.6 (341)	χ^2^(1,1916) = 1.06 p = 0.302
Unexplained pain	43.7 (448)	44.2 (481)	χ^2^(1,2113) = 0.05 p = 0.816	45.7 (575)	41.5 (322)	χ^2^(1,2033) = 3.51 p = 0.061	43.7 (791)	44.7 (135)	χ^2^(1,2112) = 0.11 p = 0.746	42.1 (499)	46.4 (340)	χ^2^(1,1918) = 3.36 p = 0.067
Unexplained weight loss	40.9 (418)	43.7 (477)	χ^2^(1,2113) = 1.62 p = 0.203	47.4 (595)	35.4 (275)	χ^2^(1,2033) = 28.14 p < 0.001	42.9 (776)	39.4 (119)	χ^2^(1,2112) = 1.28 p = 0.259	43.8 (519)	40.2 (294)	χ^2^(1,1917) = 2.45 p = 0.118
Cough or hoarseness	37.2 (381)	30.9 (337)	χ^2^(1,2114) = 9.51 p = 0.002	36.2 (455)	29.7 (231)	χ^2^(1,2034) = 9.18 p = 0.002	33.7 (610)	36.6 (111)	χ^2^(1,2113) = 0.99 p = 0.319	33.9 (402)	35.7 (261)	χ^2^(1,1918) = 0.62 p = 0.431
Difficulty swallowing	33.8 (345)	34.2 (372)	χ^2^(1,2110) = .003 p = 0.858	37.8 (474)	28.4 (220)	χ^2^(1,2030) = 19.02 p < 0.001	35.6 (644)	24.9 (75)	χ^2^(1,2109) = 13.15 p < 0.001	34.6 (410)	33.8 (247)	χ^2^(1,1915) = 0.12 p = 0.732
Sore that does not heal	27.5 (280)	25.5 (277)	χ^2^(1,2106) = 1.02 p = 0.312	28.7 (359)	23.5 (182)	χ^2^(1,2026) = 6.37 p = 0.012	25.8 (466)	29.1 (88)	χ^2^(1,2105) = 1.45 p = 0.229	24.9 (294)	27.7 (202)	χ^2^(1,1910) = 1.86 p = 0.173

Notes: *Pearson’s χ^2^ test for 2x2 tables (i.e., Yes vs. No/Don’t know for each demographic variable). Statistically significant associations at the p < 0.05 level are emboldened. ^#^Black and Minority Ethnic. ^†^Scottish Index of Multiple Deprivation.

The mean number of recognised cancer warning signs was 4.0 (SD = 2.11) out of 9. Adolescents who knew someone with cancer had significantly higher recognition than those who did not (Yes: M = 4.3, SD = 2.09 vs No: M = 3.6, SD = 2.03; t(2044) = -7.621, p < 0.001). White adolescents recognised significantly more warning signs than those from BME groups (White: M = 4.1, SD = 2.09 vs BME: M = 3.6, SD = 2.11; t(2125) = 3.801, p < 0.001). The number of warning signs recognised did not differ significantly by gender or deprivation (Girls: M = 4.1, SD = 2.02 vs Boys: 4.0, SD = 2.18; SIMD Q1: 4.0, SD = 2.15 vs SIMD Q2-5: 4.1, SD = 2.07).

Poisson regression analysis identified that adolescents who knew someone with cancer recognised 1.2 times as many cancer warning signs as those who didn’t know someone with cancer and that BME adolescents recognised significantly fewer cancer warning signs (Table 3).

**Table 3:** **Poisson regression model**: **cancer warning signs**

		95% CI		
Variable	IRR ^†^	Lower	Upper	p
Intercept	3.78	3.59	3.98	<0.001
*Ethnicity*				
BME	0.92	0.86	0.99	0.023
White	-	-	-	-
*Knew someone with cancer*				
Yes	1.19	1.13	1.25	<0.001
No	-	-	-	-
*SIMD*				
Quintile 1 (most deprived)	0.95	0.91	1.00	0.043
Quintiles 2-5	-	-	-	-

Notes: ^†^Incidence Rate Ratio.

### Barriers to help seeking

Emotional barriers were the most commonly endorsed, followed by family, service and practical barriers. Over two thirds of adolescents were ‘worried about what the doctor would find’ (71.7%) and over half were ‘scared’ (57.2%). Almost half were ‘not confident to talk about symptoms’ (48.2%) or ‘embarrassed’ (47.7%). Over a third stated they ‘would not want family to find out’ (35.8%). Over a quarter of adolescents endorsed the service barriers ‘difficult to talk to the doctor’ (29.8%) or ‘worry about wasting the doctor’s time’ (29.1%) and just under a quarter stated they would find it ‘difficult to make appointment’ (22.5%). A fifth of adolescents endorsed the practical barriers being ‘too busy’ (19.9%) or having ‘other things to worry about’ (19.2%). ‘Difficult to arrange transport’ was the least reported barrier to help seeking (14.3%). Differences in endorsement of barriers to help seeking by gender, knowing someone with cancer, ethnicity and deprivation are shown in Table 4.

**Table 4:** **Barriers to help seeking**

Barrier	Gender (n = 2,134)			Knew someone with cancer (n = 2,049)			Ethnicity (n = 2,132)			Deprivation (SIMD ^†^) (n = 1,944)		
% Yes (n)	Male (n = 1,032)	Female (n = 1,102)	Significance*	Yes (n = 1,266)	No (n = 783)	Significance*	White (n = 1,826)	BME ^#^(n = 306)	Significance*	Q1 (n = 1,205)	Q2-5 (n = 739)	Significance*
Emotional												
Worried about what the doctor might find	65.0 (639)	78.3 (837)	χ^2^(1, 2052) = 44.81, p < 0.001	74.5 (905)	68.0 (516)	χ^2^(1, 1973) = 9.98, p = 0.002	72.7 (1274)	66.1 (197)	χ^2^(1, 2051) = 5.42, p = 0.002	72.7 (832)	71.8 (515)	χ^2^(1, 1862) = 0.15, p = 0.694
Too scared	45.8 (452)	68.2 (732)	χ^2^(1, 2059) = 105.3, p < 0.001	61.1 (750)	51.7 (390)	χ^2^(1,1982) = 16.72,p < 0.001	58.0 (1023)	53.0 (157)	χ^2^(1, 2059) = 2.58, p = 0.109	56.1 (646)	59.1 (424)	χ^2^(1, 1869) = 1.69, p = 0.194
Not confident to talk about symptoms	41.4 (404)	54.7 (578)	χ^2^(1, 2032) = 36.15, p < 0.001	49.2 (590)	46.2 (349)	χ^2^(1, 1955) = 1.72, p = 0.190	48.4 (839)	46.5 (139)	χ^2^(1, 2031) = 0.39, p = 0.533	47.2 (534)	49.9 (356)	χ^2^(1, 1844) = 1.29, p = 0.256
Too embarrassed	37.8 (370)	57.5 (612)	χ^2^(1, 2044) = 79.83, p < 0.001	49.2 (597)	45.9 (346)	χ^2^(1, 1967) = 2.06, p = 0.151	48.1 (841)	46.4 (137)	χ^2^(1, 2044) = 0.27, p = 0.601	46.9 (536)	47.5 (388)	χ^2^(1, 1855) = 0.08, p = 0.774
Family												
I wouldn’t want my family to find out	34.3 (336)	37.4 (395)	χ^2^(1, 2035) = 2.20, p = 0.138	36.7 (442)	34.0 (257)	χ^2^(1, 1959) = 1.53, p = 0.217	36.3 (631)	33.1 (98)	χ^2^(1, 2034) = 1.13, p = 0.289	37.6 (427)	32.5 (232)	χ^2^(1, 1848) = 4.93, p = 0.026
Service Barriers												
Difficult to talk to doctor	25.6 (247)	34.1 (358)	χ^2^(1, 2014) = 17.41, p < 0.001	30.9 (368)	28.3 (211)	χ^2^(1, 1938) = 1.47, p = 0.226	30.4 (523)	26.4 (78)	χ^2^(1, 2014) = 1.91, p = 0.167	30.2 (340)	28.4 (200)	χ^2^(1, 1829) = 0.68, p = 0.408
Worried about wasting the doctor’s time	25.8 (251)	32.6 (342)	χ^2^(1, 2022) = 11.09, p = 0.001	29.8 (357)	27.8 (208)	χ^2^(1, 1947) = 0.81, p = 0.368	30.0 (520)	24.4 (71)	χ^2^(1, 2022) = 3.83, p = 0.050	28.8 (324)	28.3 (201)	χ^2^(1, 1836) = 0.05, p = 0.830
Difficult to make an appointment	22.5 (217)	22.4 (235)	χ^2^(1, 2014) = 0.00, p = 0.983	23.4 (279)	21.3 (158)	χ^2^(1, 1937) = 1.16, p = 0.282	21.6 (372)	27.9 (82)	χ^2^(1, 2014) = 5.64, p = 0.018	22.5 (253)	21.2 (149)	χ^2^(1, 1829) = 0.44, p = 0.506
Practical Barriers												
Too busy	20.6 (202)	19.5 (206)	χ^2^(1, 2038) = 0.44, p = 0.506	19.5 (234)	20.6 (156)	χ^2^(1, 1960) = 0.36, p = 0.548	19.2 (334)	23.8 (71)	χ^2^(1, 2037) = 3.41, p = 0.065	19.6 (223)	20.3 (144)	χ^2^(1, 1849) = 0.15, p = 0.695
Other things to worry about	16.4 (160)	22.2 (235)	χ^2^(1, 2034) = 10.84, p = 0.001	18.9 (226)	20.2 (153)	χ^2^(1, 1956) = 0.52, p = 0.472	18.8 (327)	22.5 (67)	χ^2^(1, 2033) = 2.15, p = 0.142	18.9 (214)	18.7 (133)	χ^2^(1, 1845) = 0.00, p = 0.948
Difficult to arrange transport	16.6 (162)	12.1 (127)	χ^2^(1, 2027) = 8.54, p = 0.003	12.7 (152)	16.3 (123)	χ^2^(1, 1950) = 4.79, p = 0.029	14.0 (242)	16.2 (48)	χ^2^(1, 2026) = 1.02, p = 0.312	15.5 (176)	12.7 (90)	χ^2^(1, 1840) = 2.83, p = 0.092

Notes: *Pearson’s χ^2^ test for 2x2 tables (i.e., Yes vs. No/Don’t know for each demographic variable). Statistically significant associations at the p < 0.05 level are emboldened.

The mean number of barriers to help seeking was 3.8 (SD = 2.47) out of 11. Girls endorsed a statistically significantly greater number of barriers than boys (Girls: M = 4.2, SD = 2.39 vs Boys: M = 3.3, SD = 2.47; t(2126) = -8.493, p < 0.001). The number of barriers to help seeking endorsed did not differ significantly by knowing someone with cancer, ethnicity and deprivation (Yes: M = 3.9, SD = 2.42 vs No: M = 3.7, SD = 2.52; White: M = 3.8, SD = 2.47 vs BME M = 3.7, SD = 2.48; SIMD Q1: M = 3.8, SD = 2.51 vs SIMD Q2-5: M = 3.8 SD = 2.41).

Poisson regression analysis identified that girls endorsed 1.2 times as many barriers to help seeking as boys and that higher levels of anxiety and recognition of more cancer warning signs were significantly associated with endorsing slightly more barriers to help seeking (Table 5).

**Table 5:** **Poisson regression model**: **barriers to help seeking**

		95% CI		
Variable	IRR ^†^	Lower	Upper	p
Intercept	2.41	2.25	2.58	<0.001
Anxiety	1.03	1.03	1.04	<0.001
Cancer warning signs	1.03	1.02	1.04	<0.001
*Gender*				
Female	1.24	1.18	1.30	<0.001
Male	-	-	-	-

Notes: ^†^Incidence Rate Ratio.

## Discussion

The study shows that cancer awareness among adolescents is low, confirming findings of previous investigations conducted in young people [15, 16, 45–48]. The study contributes to the evidence base by showing variation in awareness of cancer warning signs among different groups of adolescents. The study for instance, shows that adolescents from BME backgrounds recognised fewer cancer warning signs than White adolescents. This difference corroborates research among adults, suggesting the need for cultural awareness and sensitivity in interventions to raise cancer awareness [49, 50]. The study also found that girls reported a higher number of barriers to seeking medical help about cancer than boys and that ‘knowing someone affected by cancer’ influences an individual’s awareness of cancer warning signs and barriers to seeking medical help. Contextual factors (for example, ethnicity, gender and knowing someone with cancer) are therefore important factors for explaining variation in cancer awareness and barriers to seeking medical help during adolescence.

The study is unable to show the extent to which low awareness of cancer impacts seeking medical help about cancer during adolescence and uncertainty remains about the pattern of the relationship between cancer awareness and actual (as opposed to intention or number of perceived barriers) help seeking. There are however, several reasons why it is reasonable to hypothesise a relationship between low awareness of signs and symptoms of cancer and delays in seeking medical help. First, empirical evidence suggests that there is an association between identification of signs and symptoms of cancer and help seeking intentions [11, 21, 25, 51]. Second, lack of awareness or understanding of symptoms has been reported as a barrier to help seeking in young people for other conditions such as, mental health disorders [52]. Third, theoretical models of illness behaviour in response to symptoms acknowledge the role of awareness of symptoms [28]. Taken together, this body of work suggests that awareness of signs and symptoms of cancer is an important factor for promoting early presentation.

A central aim of this study was to examine whether anxiety is associated with endorsed barriers to seeking medical help about cancer. The study found that anxiety was a significant independent predictor of the number of endorsed barriers to help seeking, suggesting that the more anxious a young person is the more barriers to seeking medical help about cancer they will endorse. The reasons for this are unclear but it may be that anxious people elicit negative emotional representations of signs and symptoms of cancer and these representations of illness influence the quantity and type of perceived barriers to seeking medical help to regulate the health threat (cancer) and to regulate illness anxiety [35].

Whether anxious people actually delay seeking medical help about cancer or simply perceive more barriers to help seeking is not known and requires further investigation. Fifty years ago, Antonovsky and Hartman in a review of delay in cancer determined that both low *and* high levels of anxiety were associated with longer delay times [53]. Nevertheless, there remains a level of uncertainty about the pattern of the relationship between anxiety and help seeking. Ristvedt and Trinkaus found that a tendency toward low trait anxiety, worry and negative emotions measured using the Harm Avoidance Scale of the Temperament and Character Inventory was associated with delays in seeking medical help for symptoms of rectal cancer but found no relationship between these tendencies and help seeking when measured using the State-Trait Anxiety Inventory [24, 54, 55]. Drawing on their own and other work, Watson and Pennebaker suggest that negative affectivity (which incorporates anxiety) is not associated with more visits to the doctor but instill a note of caution because they did not examine every medical condition [56]. Most research among adolescents focuses on help seeking behaviour *for* anxiety rather than examining the impact of mental health on help seeking behaviour for other illness [57]. Given that one in ten young people experience mental health problems further research about relationships between anxiety during adolescence and help seeking behaviour for different types of illness, including cancer is warranted [58].

The study replicates and strengthens the findings of a previous study showing that most endorsed barriers are emotional as opposed to service or practical barriers [15]. Cancer is feared more than any other life-threatening condition and fear of cancer (an emotional representation of the disease) may therefore explain why adolescents cite emotional barriers such as being ‘worried about what the doctor might find’ to seeking medical help about cancer [59]. Endorsement of emotional barriers are in line with research showing that feelings are often more persuasive in health decision-making processes compared to rational processes [60–62]. There is a growth of empirical support for what Slovic and colleagues refer to as the ‘affect heuristic’ in studies of the behavioural decision-making process for cancer prevention [63]. This work suggests that affective beliefs about risk are stronger predictors of intentions and health behaviours than cognitively based beliefs across a range of health behaviours including exposure to ultra-violet radiation from sunbed use and sunbathing, diet, smoking and physical activity [64–67].

### Limitations

Our study has several limitations. First, there is inevitably a limit to the generalisability of these findings beyond this sample, which was drawn exclusively from schools in Glasgow. The average deprivation experienced by adolescents living in Glasgow was higher than that found in the UK as a whole [68]. Moreover, the so-called ‘Glasgow Effect’ , which is a term used to describe the higher levels of mortality and poor health experienced in Glasgow over and above that explained by its socio-economic profile, may have influenced the results [68]. Second, only a limited range of independent variables were available to construct the most parsimonious Poisson regression models. There are likely other factors that explain adolescent cancer awareness and barriers to seeking medical help that have not been measured in this study. Other research provides potential additional independent variables for inclusion in future investigations, such as, attitudes towards help seeking, risk perception, anticipated regret, emotional regulation, and personal confidence and self efficacy in seeking medical help [25, 26, 35]. Third, this study examined adolescents’ *perceptions* of barriers to help seeking, which may not be the same as *actual* help seeking behaviour. Moreover, while we are able to report the proportion of adolescents endorsing particular barriers to help seeking we are not able to report if there is an actual difference in medical help seeking between those who endorse an emotional barrier and those who do not. Retrospective accounts of the experience of cancer diagnosis found that young people with cancer did not purposefully delay seeking help through fear [69]. Thus, further research about the relationship between illness perceptions of cancer and actual help seeking behaviour is required. Fourth, even in the context of a survey with a relatively high response rate, there is potential for non-response bias. Finally, we are unable to report whether the differential patterning of cancer awareness observed in this study reflects a continuation of childhood patterns i.e. the ‘childhood persistence pattern’ or emerges in adolescence i.e. the ‘adolescent emergent pattern’ [70]. Moreover, this study focused on early adolescence (12/13 year olds), during which different patterns of cancer awareness than late adolescent and early adulthood (i.e. 15–24 year olds) may exist. Understanding when differences emerge may be important for the purposes of intervening to change public cancer awareness and help seeking behaviour. Future longitudinal studies are therefore required to contribute towards understanding how cancer awareness and medical help seeking change across the life-course.

## Conclusions

Adolescence ‘*presents a window of opportunity to set the stage for healthy and productive adulthood and to reduce the likelihood of problems in the years that lie ahead*’ [71]. Improving cancer awareness and help seeking behaviour during adolescence may therefore contribute to improvement across the life-course. Awareness of signs and symptoms of cancer is low and barriers to seeking medical help are high in this age group and are influenced by contextual (for example, ethnicity, gender, knowing someone with cancer), and emotional (for example, anxiety, fear, worry) factors. Thus, interventions to increase cancer awareness in schools are required and may address the predominantly adult focus of cancer awareness campaigns. Such interventions to improve public cancer awareness and help seeking about cancer may benefit from having a more emotional focus by, for example, including references to feelings, such as, fears and worries about getting cancer instead of, or in addition to, cognition-related terms such as, talking about the probability of getting cancer.
